# Long-term antibody response after the third dose of inactivated SARS-CoV-2 vaccine in MASLD patients

**DOI:** 10.1186/s12876-024-03402-9

**Published:** 2024-09-30

**Authors:** Jin Cui, Lianbang Wang, Armin Ghavamian, Xuemei Li, Gongzheng Wang, Tao Wang, Min Huang, Qi Ru, Xinya Zhao

**Affiliations:** 1 Department of Radiology, Shandong Provincial Hospital Affiliated to Shandong First Medical University, Jinan, Shandong 250021 China; 2 Department of Radiology, Shandong Provincial Hospital, Cheeloo College of Medicine, Shandong University, Jinan, Shandong 250021 China; 3 Department of Gastroenterology, Shandong Provincial Hospital, Shandong University, Jinan, Shandong 250021 China; 4 Department of Laboratory, Shandong Provincial Hospital Affiliated to Shandong First Medical University, Jinan, Shandong 250021 China; 5 Department of Ultrasound, Qilu Hospital (Qingdao), Cheeloo College of Medicine, Shandong University, 758 Hefei Road, Qingdao, Shandong 266035 China; 6 Key Laboratory of Endocrine Glucose & Lipids Metabolism and Brain Aging, Ministry of Education, Jinan, Shandong 250021 China

**Keywords:** Metabolic dysfunction-associated steatotic liver disease, SARS-CoV-2, Vaccine, Antibody response

## Abstract

**Background:**

Metabolic dysfunction-associated steatotic liver disease (MASLD) patients are at an elevated risk of developing severe coronavirus disease 2019 (COVID-19). The objective of this study was to assess antibody responses and safety profiles six months after the third dose of the inactivated acute respiratory syndrome coronavirus 2 (SARS-CoV-2) vaccine in MASLD patients.

**Methods:**

This study included MASLD patients and healthy volunteers without a history of SARS-CoV-2 infection. Blood samples were collected six months after receiving the third dose of the inactivated vaccine to measure the levels of neutralizing antibodies (NAbs) and anti-spike IgG antibodies against SARS-CoV-2.

**Results:**

A total of 335 participants (214 MASLD patients and 121 healthy volunteers) were enrolled. The seroprevalence of NAb was 61.7% (132 of 214) in MASLD patients and 74.4% (90 of 121) in healthy volunteers, which was a significant difference (*p* = 0.018). Statistically significant differences in IgG seroprevalence were also observed between MASLD patients and healthy volunteers (*p* = 0.004). Multivariate analysis demonstrated that the severity of MASLD (OR, 2.97; 95% CI, 1.32–6.68; *p* = 0.009) and age (OR, 1.03; 95% CI, 1.01–1.06; *p* = 0.004) were independent risk factors for NAb negativity in MASLD patients. Moderate/severe MASLD patients had a lower NAb seroprevalence than mild MASLD patients (45.0% vs. 65.5%, *p* = 0.016).

**Conclusion:**

Lower antibody responses were observed in MASLD patients six months after their third dose of the inactivated vaccine than in healthy volunteers, providing further assistance in monitoring patients who are more vulnerable to hypo-responsiveness to SARS-CoV-2 vaccines.

**Supplementary Information:**

The online version contains supplementary material available at 10.1186/s12876-024-03402-9.

## Introduction

The advent of the severe acute respiratory syndrome coronavirus 2 (SARS-CoV-2) in late 2019 led to the onset of the coronavirus disease 2019 (COVID-19) pandemic, an unprecedented public health crisis that has resulted in over 760 million infections and 6.9 million deaths globally [1]. Even if COVID-19 pandemic is waning at present due to the developed immunity in human population, SARS-CoV-2 may emerge as a recurrent and seasonal pathogen [2]. Metabolic dysfunction-associated steatotic liver disease (MASLD), with a global prevalence estimated at approximately 30%, is presently recognized as a multisystem disorder that exerts deleterious effects not only on hepatic tissues but also on various extrahepatic organs [3–6]. During the COVID-19 pandemic, MASLD patients were at an elevated risk of developing severe COVID-19 [7–10]. Vaccination is critical to the containment of the epidemic and the reduction of overall mortality and is protective against SARS-CoV-2 infection and exacerbation of COVID-19 [11]. The immune response to SARS-CoV-2 infection may be compromised by chronic inflammation induced by oxidative stress in the context of MASLD, leading to increased viral loads and accelerated disease progression [12, 13]. Therefore, it is necessary to investigate the immunogenicity of SARS-CoV-2 vaccination in MASLD patients.

Neutralization assays and anti-spike IgG antibody detection had been widely used to study humoral immune responses to SARS-CoV-2 [14, 15]. In patients with chronic liver disease resulting from various etiologies, MASLD patients exhibited a decreased antibody response after receiving two doses of the inactivated SARS-CoV-2 vaccine [16]. The humoral response induced by the third dose of vaccination significantly contributed to immune protection against severe outcomes and mortality associated with COVID-19 [17, 18]. Chinese authorities approved the administration of a third dose (booster dose) of the SARS-CoV-2 vaccine in September 2021. The immune response was indeed improved after the third dose of SARS-CoV-2 vaccination both in terms of humoral immunity and cellular immunity [19]. It should be noted that antibody levels gradually decrease and the risk of breakthrough infection increases over time [20, 21], and a decrease in immunity was observed six months after vaccination [22, 23]. However, there were no published data regarding long-term immune responses after the third dose of the inactivated SARS-CoV-2 vaccine in MASLD patients.

Therefore, the objective of this present study was to assess the antibody responses and safety profile six months after the third dose of the inactivated SARS-CoV-2 vaccine in MASLD patients. Additionally, risk factors for negative responses to vaccination were identified in this highly vulnerable patient population.

## Methods

### Participants

In this prospective single institutional study, patients with MASLD and healthy volunteers were recruited at Shandong Provincial Hospital. All participants in this study completed three doses (two priming doses and one booster dose) of inactivated SARS-CoV-2 vaccination (BBIBP-CorV or CoronaVac) between June 2021 to September 2021 according to the standard protocol.

**Fig. 1:**
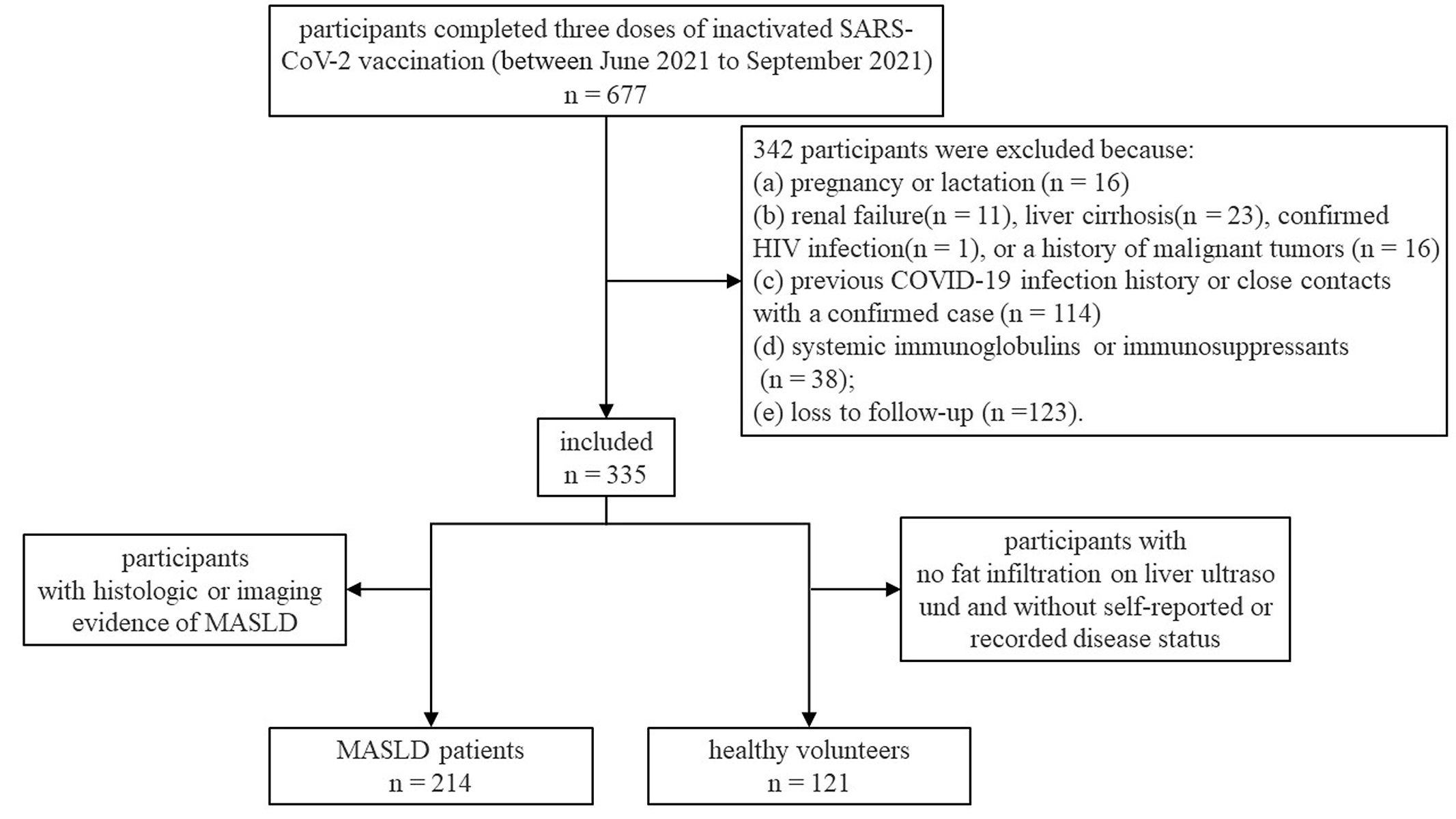
Flowchart of the study. COVID-19, coronavirus disease 2019; MASLD, metabolic dysfunction-associated steatotic liver disea; SARS-CoV-2, severe acute respiratory syndrome coronavirus 2

Adult patients (aged ≥ 18 years) with histologic or imaging evidence of MASLD were recruited. The inclusion criteria for healthy volunteers were no fat infiltration on liver ultrasound and no self-reported or recorded disease status. The exclusion criteria for all participants included: (a) pregnancy or lactation; (b) renal failure, liver cirrhosis, confirmed HIV infection, or a history of malignant tumors; (c) previous COVID-19 infection history or close contacts with a confirmed case; (d) systemic immunoglobulins or immunosuppressants; and (e) loss to follow-up. Liver cirrhosis was defined by the presence of either histological evidence of cirrhosis on liver biopsy or radiological findings consistent with cirrhosis [24]. The flowchart of the study was shown in Fig. 1.

The severity of liver steatosis is evaluated by abdominal ultrasound, which has been demonstrated to have acceptable sensitivity for the detection of fatty liver [25]. In general, steatosis on ultrasonography is determined by at least two of these features: enhanced liver brightness, poor visualization of intrahepatic structures (after the exclusion of excessive alcohol abuse and other liver diseases), diffuse enhancement of liver-kidney contrast agents and deep attenuation. The severity of MASLD is divided into three levels: mild (diffuse increase in fine echoes in liver parenchyma), moderate (diffuse increase in fine echoes with impaired visualization of the intrahepatic vessel borders and diaphragm), and severe (diffuse increase in fine echoes with non-visualization of the intrahepatic vessel borders and diaphragm) [26].Approval for the study was obtained from the hospital’s ethics committee, and written informed consent was obtained from all participants before the commencement of the study.

### Data collection

From November 2021 to March 2022, blood samples for measuring antibodies were collected from all participants six months after receiving their third dose of the inactivated vaccine. Baseline characteristics, clinical data, and laboratory test results were collected from the patients’ electronic medical records and through interviews. Vaccine-related details, including the vaccine manufacturer and the time of vaccination, were obtained using a structured questionnaire.

### Evaluation of antibody responses

A chemiluminescence immunoassay (Maccura Biotechnology Co., Ltd., China) was used to evaluate NAb and anti-spike IgG antibodies six months after three doses of the inactivated vaccine. The detection principle was as follows: first, blood samples are coated with the coronavirus RBD antigen and streptavidin magnetic particles, and NAb/IgG in the sample binds to the RBD-binding antigen to form an immune complex. Second, the acridine ester-labelled ACE2 antigen was added, the sample was washed and mixed with substrate solution for chemiluminescence, and the luminescence signal was measured. The luminescence signal value was negatively correlated with the NAb/IgG content in the samples. The NAb concentrations were quantified in arbitrary units per milliliter (AU/mL), where AU/mL > 6.00 was considered positive. Anti-spike IgG levels were presented as the signal-to-cut-off ratio (S/CO), where S/CO > 1.00 was considered positive. When the NAb concentration was lower than the detection limit (3.00 AU/mL), a value of 2.00 AU/mL was assigned.

### Adverse events monitoring

The adverse events reported by the participants were collected using a questionnaire, reviewed by the investigators, and scored using the scale published by the National Medical Products Administration of China (Edition 2019).

### Statistical analysis

The analyses were conducted using SPSS (version 25.0.0, IBM). Appropriate methods corresponding to the data type were used for statistical analysis. Continuous variables were presented either as medians with interquartile ranges (IQRs) or means with standard deviations, whereas categorical variables were presented as numerical values and percentages (%). The statistical significance between groups was assessed for continuous variables using either ANOVA or the Mann‒Whitney U test and for categorical variables using either the chi‒square test or the continuity-corrected chi‒square test. Correlations between variables were tested by Spearman correlation. We used logistic regression to adjust for factors that differed significantly between MASLD patients and healthy volunteers when comparing immunogenic outcomes. Univariate and multivariate analyses were conducted to identify risk factors for antibody negativity using logistic regression models. These results were reported as odds ratios (ORs) and 95% confidence intervals (CIs). Statistical significance was defined as a two-tailed *p* value < 0.05. GraphPad Prism 9 (GraphPad Software, Inc.) was used for plotting. In all the graphs, the significance levels were *p ≤ 0.05 and **p ≤ 0.01.

## Results

### Characteristics of participants

A total of 335 participants (214 MASLD patients and 121 healthy volunteers) were enrolled. The participants were monitored by polymerase chain reaction and no participant tested positive for SARS-CoV-2 infection during the follow-up period. For the safety analysis, 334 questionnaires were completed by 335 participants. The demographic characteristics and vaccination-related details of both groups were shown in Table 1. The MASLD patients included 148 (69.2%) males and 66 (30.8%) females. The proportion of males among the MASLD patients was greater than that among the healthy volunteers (69.2% vs. 37.2%; *p* < 0.001). The median age (42.5 vs. 45.5 years, *p* = 0.054) was similar between the two groups. A greater percentage of MASLD patients were overweight or obese than were healthy volunteers (79.4% vs. 33.1%; *p* < 0.001). Moreover, the incidence of liver function abnormalities was significantly elevated in MASLD patients (*p* < 0.001). The time from the third dose to blood collection was comparable between MASLD patients and healthy volunteers (*p* = 0.076).

**Table 1:** Baseline characteristics of MASLD patients and healthy volunteers

	Whole population	MASLD patients	Healthy controls	*P* value
	(*N* = 335)	(*N* = 214)	(*N* = 121)	
Age^a^, y	45.0 (35.0, 56.0)	45.5 (35.0, 58.0)	42.5 (35.0, 52.0)	0.054
Gender, (male, n (%))	193 (57.6)	148 (69.2)	45 (37.2)	< 0.001
BMI^a^, Kg/m²	24.9 (22.7, 27.4)	26.1 (24.2, 28.4)	22.3 (20.8, 24.6)	< 0.001
Overweight or obesity, n (%)	210 (62.7)	170 (79.4)	40 (33.1)	< 0.001
Time from 3rd vaccination^a^	196.0 (165.0, 242.0)	200.0 (165.0, 245.0)	183.0 (165.0, 225.0)	0.076
PLT^a^, 10^9^	253.5(216.0,291.3)	249.5 (211.8, 288.0)	259.0 (223.3, 297.5)	0.082
WBC^a^,10^9^	6.1 (5.2, 7.1)	6.3 (5.3, 7.3)	5.8 (5.1, 6.9)	0.008
AST^a^, U/L	22.0 (18.0, 27.0)	24.0 (20.0, 29.0)	19.5 (17.0, 24.0)	< 0.001
ALT^a^, U/L	20.0 (14.0, 31.0)	24.5 (16.8, 39.3)	15.0 (11.0, 21.0)	< 0.001
GGT^a^, U/L	25.0 (17.0, 41.3)	31.0 (22.0, 50.3)	17.0 (14.0, 25.8)	< 0.001
AKP^a^, U/L	74.0 (63.0, 88.3)	79.5 (66.8, 96.0)	66.0 (57.0, 80.8)	< 0.001
ALB^b^, g/L	44.6 (2.3)	44.6 (2.5)	44.5 (2.1)	0.115
TBIL^a^, mmol/L	14.0 (10.7, 18.2)	14.4 (11.1, 19.0)	13.5 (10.0, 16.7)	0.017
DBIL^a^, mmol/L	2.5 (1.9, 3.2)	2.6 (1.9, 3.3)	2.4 (1.8, 2.9)	0.048
BUN^a^, mmol/L	4.9 (4.0, 5.8)	4.9 (4.1, 5.9)	4.6 (3.9, 5.4)	0.065
Cr^b^, µmol/L	69.5 (13.5)	71.2 (13.7)	66.4 (12.6)	0.399
GLU^a^, mmol/L	5.0 (4.7,5.5)	5.2 (4.8,5.7)	4.8 (4.6,5.2)	< 0.001
TC^b^, mmol/L	5.0 (1.0)	5.1 (1.0)	4.9 (0.9)	0.074
TG^a^, mmol/L	1.4 (1.0, 1.9)	1.6 (1.2, 2.4)	1.1 (0.7, 1.4)	< 0.001
LDL^a^, mmol/L	3.1 (0.8)	3.2 (0.8)	2.9 (0.7)	0.126
HDL^a^, mmol/L	1.4 (1.2,1.6)	1.3 (1.1,1.5)	1.6 (1.4,1.7)	< 0.001
Comorbidities, n (%)				
CAD, n (%)	16 (4.8)	12 (5.6)	4 (3.3)	0.495
HTN, n (%)	24 (7.2)	12 (5.6)	12 (9.9)	0.142
DM, n (%)	25 (7.5)	23 (10.7)	2 (1.7)	< 0.001
UA, µmol/L	360.1 (89.4)	380.2 (89.8)	325.3 (77.4)	0.104
Fibrosis (fib-4 score > 1.3), n (%)	72 (21.5)	56 (26.2)	16 (13.2)	0.006

*Notes*^a^Presented as median (interquartile range). ^b^Presented as mean (standard deviation). *P* < 0.05 was considered statistically significant

*Abbreviations* AKP, alkaline phosphatase; ALB, albumin; ALT, alanine aminotransferase; AST, aspartate aminotransferase; BMI, body mass index; BUN, blood urea nitrogen; CAD, coronary artery disease; Cr, creatinine; DBIL, direct bilirubin; DM, diabetes mellitus; GGT, g-glutamyl transpeptidase; GLU, glucose; HDL, high-density lipoprotein; HTN, hypertension; LDL, low-density lipoprotein; MASLD, metabolic dysfunction-associated steatotic liver disease; PLT, platelet; TBIL, total bilirubin; TC, total cholesterol; TG, triglyceride; UA, uric acid; WBC, white blood cell

###  Long-term antibody response after the third dose of the inactivated SARS-CoV-2 vaccine in MASLD patients

**Fig. 2:**
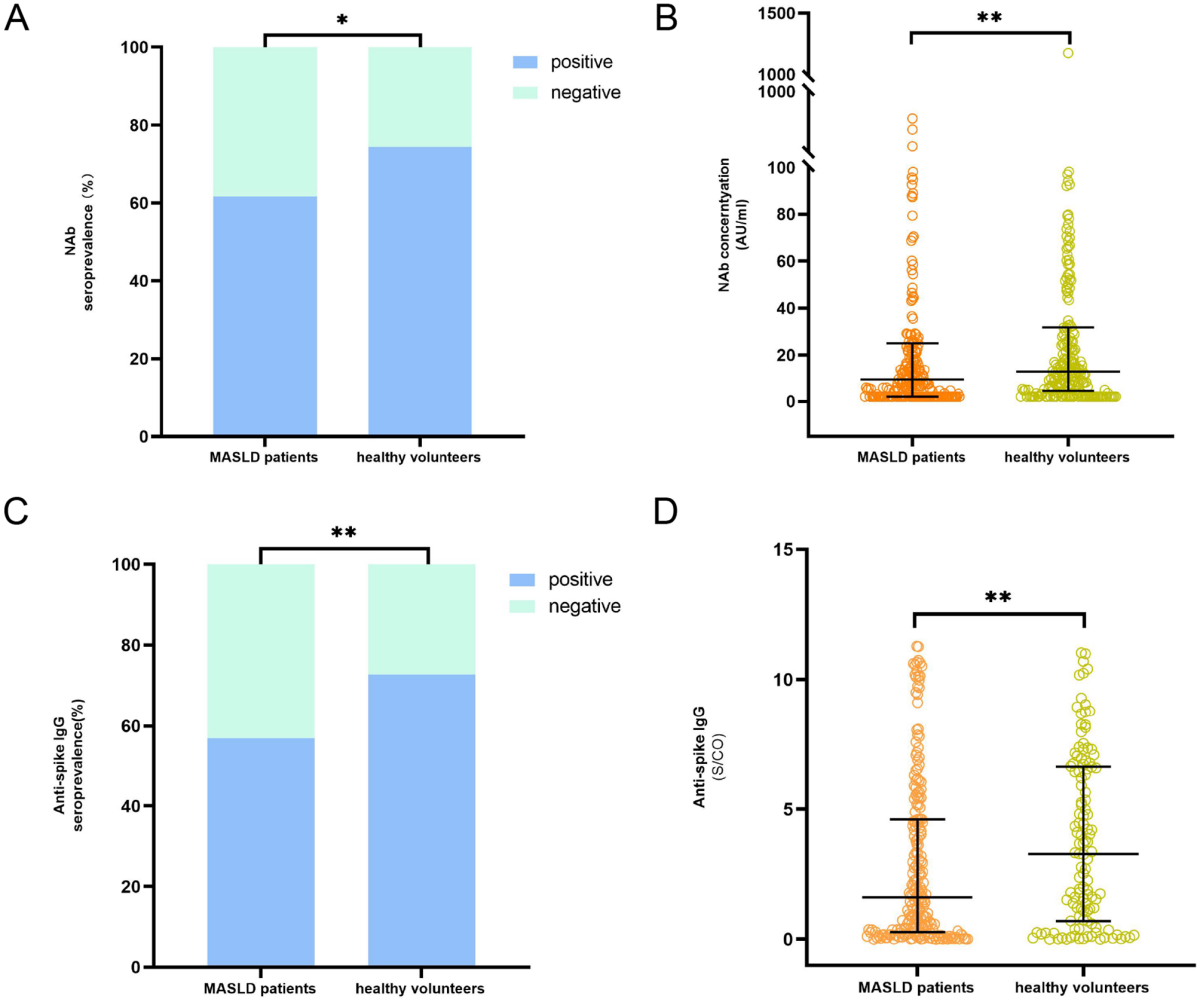
Antibody response 6 months after the third dose of inactivated SARS-CoV-2 vaccination in MASLD patients and healthy volunteers. (**A**) Seroprevalences of Nab and (**B**) NAb concentrations. (**C**) Seroprevalences of anti-spike IgG and (**D**) anti-spike IgG levels. NAb concentrations above 6.00 AU/mL were considered positive. IgG levels above 1.00 S/CO were considered positive. NAb, neutralizing antibody; MASLD, metabolic dysfunction-associated steatotic liver disease

At six months after the third dose of inactivated SARS-CoV-2 vaccination, the seroprevalences of NAb were 61.7% (132 of 214) in MASLD patients and 74.4% (90 of 121) in healthy volunteers. Statistically significant differences in NAb seroprevalence were observed between MASLD patients and healthy volunteers (*p* = 0.018). (Fig. 2A) The concentrations of NAbs were 9.32 AU/mL (IQR, 2.00-25.02 AU/mL) in MASLD patients and 15.79 AU/mL (IQR, 5.02–51.19 AU/mL) in healthy volunteers. MASLD patients exhibited significantly lower NAb concentrations than healthy volunteers (*p* = 0.007). (Fig. 2B)

At six months after the third dose of inactivated vaccine, 57.0% (99 of 174) of MASLD patients and 72.7% (88 of 121) of healthy volunteers were seropositive for anti-spike IgG. Statistically significant differences in IgG seroprevalence were also observed between MASLD patients and healthy volunteers (*p* = 0.004) (Fig. 2C). Anti-spike IgG levels were 1.61 S/CO (IQR, 0.28–4.61) in MASLD patients and 3.28 S/CO (IQR, 0.70–6.63) in healthy volunteers. Compared with healthy volunteers, MASLD patients also exhibited significantly lower IgG levels (*p* = 0.009) (Fig. 2D).

**Fig. 3:**
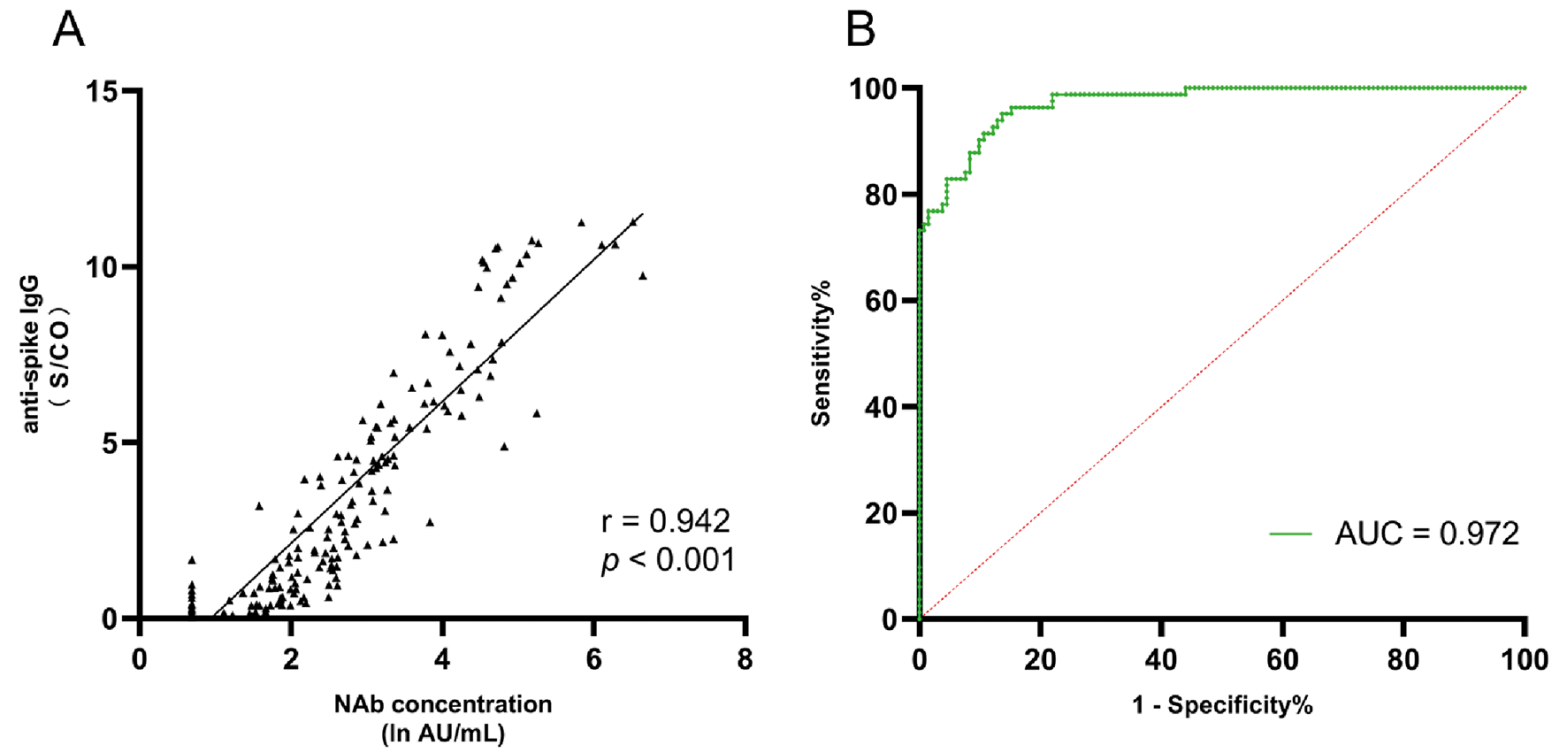
(**A**) Correlation between the levels of NAb and anti-spike IgG in MASLD patients. (**B**) Diagnostic accuracy of anti-spike IgG antibody to predict detectable NAb. NAb, neutralizing antibody; MASLD, metabolic dysfunction-associated steatotic liver disease

Furthermore, a strong correlation was observed between the NAb and anti-spike IgG levels, with a Spearman correlation coefficient of 0.942 (*p* < 0.001) in MASLD patients (Fig. 3A). Anti-spike IgG levels were higher in patients who were positive for NAbs than in those who were negative for NAbs (3.95 S/CO [IQR, 1.78–6.27] vs. 0.16 S/CO [IQR 0.06–0.40]; *p* < 0.001). The AUC of the ROC curve of anti-spike IgG for detecting Nabs was 97% (95% CI, 95–99) (*p* < 0.001) (Fig. 3B). With a cut-off point of 1.15 S/CO for anti-spike IgG, the sensitivity was 86.4%, and the specificity was 95.1%.

### Risk factors associated with the serological response after the third dose of the inactivated SARS-CoV-2 vaccine in MASLD patients

To identify risk factors related to a negative serological response to SARS-CoV-2 vaccines in MASLD patients, univariate and multivariate analyses were performed (Table 2). Considering the time from the third dose of the inactivated vaccine to sample collection, the severity of MASLD (OR = 2.97; 95% CI, 1.32–6.68; *p* = 0.009) and age (OR = 1.03; 95% CI, 1.01–1.06; *p* = 0.004) were confirmed to be independent risk factors for a negative serological response to NAb. Similar results were obtained for anti-spike IgG negativity in MASLD patients (Supplementary Table).

**Table 2:** Factors related to NAb negativity in MASLD patients

Characteristics	Univariate analysis		Multivariate analysis	
	OR (95%CI)	P Value	OR (95%CI)	P Value
Age, y	1.030 (1.012, 1.049)	0.001	1.03(1.01, 1.06)	0.004
Gender, (male, n (%))	1.572 (0.982, 2.514)	0.059		
BMI, Kg/m²	1.083 (1.017, 1.152)	0.013		
Overweight or obesity	1.753 (1.075, 2.859)	0.025		
Vaccination status				
Time from 2nd vaccination	0.995 (0.989, 1.002)	0.164	0.98 (0.97, 0.99)	0.003
Time from 3rd vaccination	1.002 (0.998, 1.006)	0.282	1.01 (1.00, 1.01)	0.033
MASLD severity (moderate/severe vs. mild)	2.444 (1.216, 4.913)	0.012	2.97 (1.32, 6.68)	0.009
PLT, 10^9^	0.999 (0.995, 1.003)	0.711		
WBC, 10^9^	1.009 (0.873, 1.166)	0.905		
AST, U/L	1.000 (0.988, 1.012)	0.985		
ALT, U/L	1.001 (0.996, 1.006)	0.718		
GGT, U/L	1.009 (1.001, 1.016)	0.018		
AKP, U/L	1.006 (0.996, 1.017)	0.231		
ALB, g/L	1.009 (0.914, 1.113)	0.862		
TBIL, mmol/L	0.981 (0.943, 1.021)	0.349		
DBIL, mmol/L	0.881 (0.700, 1.110)	0.282		
BUN, mmol/L	1.097 (0.907, 1.328)	0.339		
Cr, µmol/L	1.002 (0.985, 1.019)	0.794		
GLU, mmol/L	1.143 (0.954, 1.370)	0.147		
TC, mmol/L	0.997 (0.789, 1.259)	0.978		
TG, mmol/L	1.139 (0.908, 1.428)	0.262		
LDL, mmol/L	1.096 (0.812, 1.481)	0.548		
HDL, mmol/L	0.637 (0.315, 1.287)	0.208		
Comorbidities, n (%)				
DM, n (%)	3.344 (1.450, 7.711)	0.005		

*Abbreviations* AKP, alkaline phosphatase; ALB, albumin; ALT, alanine aminotransferase; AST, aspartate aminotransferase; BMI, body mass index; BUN, blood urea nitrogen; cr, creatinine; DBIL, direct bilirubin; DM, diabetes mellitus; GGT, g-glutamyl transpeptidase; GLU, glucose; HDL, high-density lipoprotein; LDL, low-density lipoprotein; NAb, neutralizing antibody; MASLD, metabolic dysfunction-associated steatotic liver disease; PLT, platelet; TBIL, total bilirubin; TC, total cholesterol; TG, triglyceride; WBC, white blood cell

**Fig. 4:**
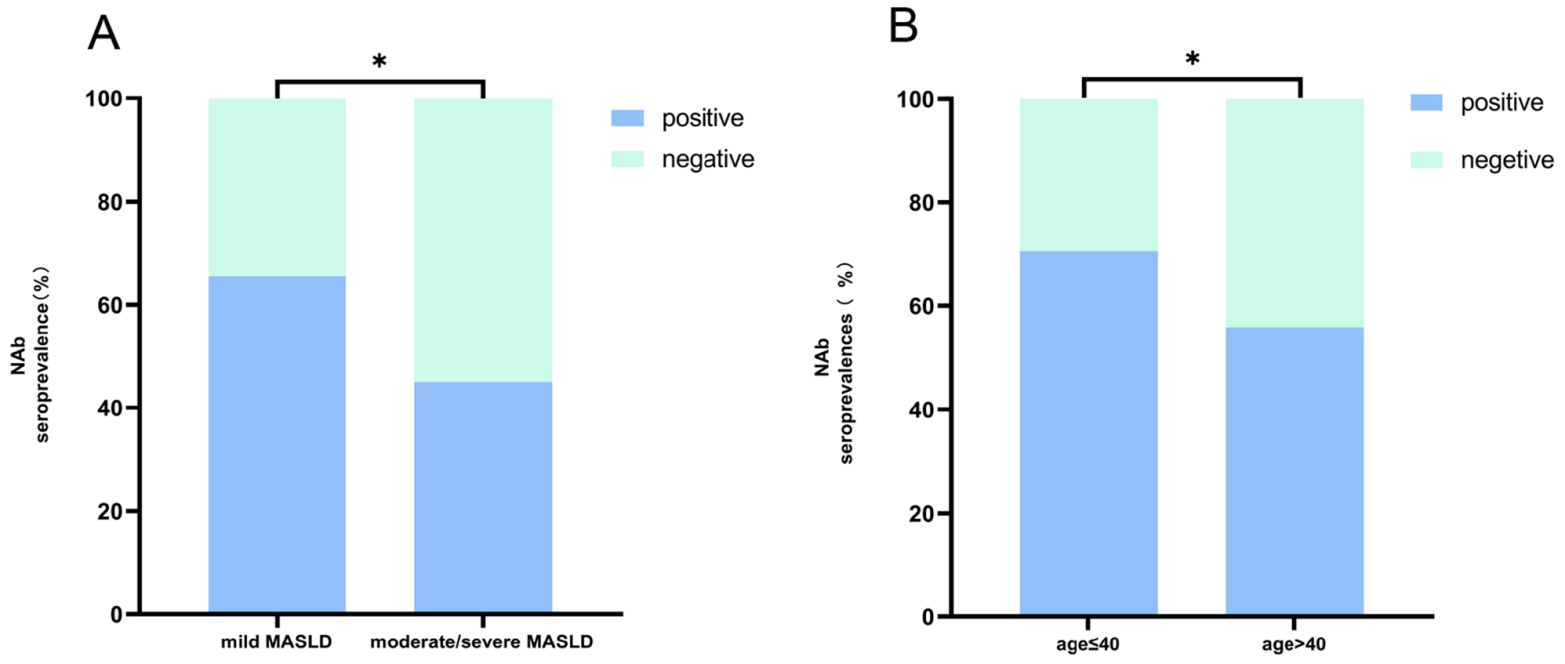
The seroprevalences of NAb stratified according to the severity of MASLD (**A**) and age (**B**), respectively. NAb, neutralizing antibody; MASLD, metabolic dysfunction-associated steatotic liver disease

NAb seroprevalences were 65.5% (114 of 174) in patients with mild MASLD and 45.0% (18 of 40) in patients with moderate/severe MASLD. The differences in the seroprevalence of NAb between patients with mild MASLD and patients with moderate/severe MASLD were statistically significant (*p* = 0.016). (Fig. 4A) The seroprevalences of NAb were 70.6% (60 of 85) in younger patients (aged ≤ 40 years) and 55.8% (72 of 129) in older patients (aged > 40 years). The differences in the seroprevalence of NAb between younger patients and older patients were statistically significant (*p* = 0.030). (Fig. 4B)

### Vaccine safety

The incidence of adverse events in MASLD patients and healthy volunteers who received three doses of the inactivated SARS-CoV-2 vaccine were shown in Table 3. The overall incidence of adverse events within 28 days was similar between MASLD patients and healthy volunteers (27.1% vs. 20.0%, *p* = 0.148). All the above adverse events were mild (grades 1 and 2) and self-limiting, and there were no serious adverse events (grades 3 and 4), such as serious thromboembolic events or myocarditis. There was no significant difference in injection site or systemic adverse reactions between MASLD patients and healthy volunteers (all *p* > 0.05). MASLD patients did not experience significant abnormal elevation of liver function markers within 28 days of booster vaccination.

**Table 3:** Adverse events after SARS-CoV-2 vaccination in MASLD patients

	Whole population (*N* = 334)	MASLD patients (*N* = 214)	Healthy controls (*N* = 120)	*p* value
Total reactions within 7 days	74 (22.2)	53 (24.8)	21 (17.5)	0.125
Total reactions within 28 days	82 (24.6)	58 (27.1)	24 (20.0)	0.148
Injection site adverse reactions				
Swelling	12 (3.6)	10 (4.7)	2 (1.7)	0.267
Induration	9 (2.7)	7 (3.3)	2 (1.7)	0.605
Itch	10 (3.0)	6 (2.8)	4 (3.3)	1.000
Pain	40 (12.0)	27 (12.6)	13 (10.8)	0.630
Redness	20 (6.0)	13 (6.1)	7 (5.8)	0.929
Systemic adverse reactions				
Appetite impaired	3 (0.9)	3 (1.4)	0 (0)	0.485
Cough	10 (3.0)	7 (3.3)	3 (2.5)	0.950
Chill	8 (2.4)	6 (2.8)	2 (1.7)	0.780
Diarrhea	9 (2.7)	5 (2.3)	4 (3.3)	0.851
Dyspnea	1 (0.3)	1 (0.5)	0 (0)	1.000
Fever	6 (1.8)	3 (1.4)	3 (2.5)	0.768
Fatigue	35 (10.5)	22 (10.3)	13 (10.8)	1.000
Headache	4 (1.2)	2 (0.9)	2 (1.7)	0.947
Hypersensitivity	2 (0.6)	2 (0.9)	0 (0)	0.538
Joint pain	10 (3.0)	6 (2.8)	4 (3.3)	1.000
Muscle pain	10 (3.0)	6 (2.8)	4 (3.3)	1.000
Nausea	6 (1.8)	2 (0.9)	4 (3.3)	0.248
Oropharyngeal pain	3 (0.9)	1 (0.5)	2 (1.7)	0.610
Vomiting	6 (1.8)	3 (1.4)	3 (2.5)	0.768
Syncope	4 (1.2)	3 (1.4)	1 (0.8)	1.000
Skin rash	10 (3.0)	7 (3.3)	3 (2.5)	0.950

*Note* Data are presented as n (%); MASLD, metabolic dysfunction-associated steatotic liver disease

## Discussion

SARS-CoV-2 is the third zoonotic beta-coronavirus to endanger the human population over the last two decade, emphasizing the possibility for future emergence of novel coronaviruses [27]. Acquiring a comprehensive comprehension of vaccine-induced immune responses against SARS-CoV-2 is of utmost importance for mitigating the impact of the post-pandemic period and preparing for future pandemics. This study assessed long-term antibody responses and safety profiles in MASLD patients after receiving a third dose of inactivated SARS-CoV-2 vaccination. Lower seroprevalences for both NAb and IgG were observed in MASLD patients six months after their third dose of the inactivated vaccine than in healthy volunteers. The severity of MASLD and age were identified as independent risk factors for NAb negativity in MASLD patients. Furthermore, moderate/severe MASLD patients had a lower NAb seroprevalence than mild MASLD patients.

As chronic liver disease is associated with a higher infection risk and greater severity of COVID-19, concerns have been raised about SARS-CoV-2 vaccine responses and potential effects. This is the first study to assess long-term antibody responses in MASLD patients, who received a third dose of inactivated SARS-CoV-2 vaccination. We found that NAb seroprevalences were lower in MASLD patients six months after the third dose of the inactivated vaccine than in healthy volunteers. This may be explained by the dysregulated immune system in MASLD, particularly the proinflammatory milieu resulting from lipid accumulation, an imbalance between lymphocyte and neutrophil counts and an imbalance within helper T-cell subsets [28–30]. Liver cirrhosis was excluded from our study, since patients with cirrhosis are known to hypo-respond to inactivated vaccines [31–33]. Short-term antibody responses have been reported, and patients with MASLD appeared to respond well after receiving two doses of SARS-CoV-2 vaccination [34]. Patients with chronic liver diseases, including liver cirrhosis, had lower short-term immunologic response and lower NAb levels after two doses of inactivated whole-virion SARS-CoV-2 vaccines [35–37]. The worldwide spread and rapid replication cycle have resulted in the emergence of several viral variants of SARSCoV-2, leading to changes in viral fitness and the capacity to evade neutralizing antibodies [38, 39]. It will be required to induct durable immunity or perform booster vaccinations periodically to protect vulnerable populations, such as MASLD patients. Our findings highlighted the significance of providing further assistance in monitoring patients who are more vulnerable to hypo-responsiveness to SARS-CoV-2 vaccines.

Our study found that the seroprevalences and levels of anti-spike IgG were lower in patients with MASLD than those in healthy volunteers six months after the third dose of inactivated SARS-CoV-2 vaccination. Previously, Simão et al. reported that IgG antibody levels decreased in patients with MASLD, cancer patients, and individuals receiving metabolic treatments after the second vaccination [16]. Huang et al. reported that the anti-SARS-CoV-2 spike IgG titers were comparable between participants with and without significant liver fibrosis, but their findings were limited by the small sample size (*n* = 45) [40]. Yang et al. found that there was no significant difference in antibody response six months after three doses of SARS-CoV-2 inactivated vaccine between patients with chronic liver disease and healthy controls, where the main etiology was hepatitis B virus infection (67.8%) but not MASLD [41]. In addition, there was a strong correlation between the levels of anti-spike IgG and NAb in this study, which is consistent with previous research [42].

We found that the severity of MASLD was an independent risk factor for NAb negativity in MASLD patients, which might be associated with the basis of immune impairment [43, 44]. Moderate/severe MASLD patients had a lower NAb seroprevalence than mild MASLD patients. It is crucial to provide valuable COVID-19 infection prevention methods and take measures such as social distancing, quarantine and isolation for moderate/severe MASLD patients. If mild MASLD patients and healthy participants were included as the control group, the study revealed no difference in seroconversion rates between the moderate/severe hepatic steatosis group and the control group after two doses of the vaccine within two months [45]. Older MASLD patients had lower NAb seroprevalences. These results could be explained in part by immunosenescence and concomitant comorbidities in older patients [46, 47].

The overall incidence of adverse events was not significantly different after the third vaccination between MASLD patients and healthy volunteers. The adverse events in both groups were not severe or self-limiting, and no MASLD patients experienced liver-related adverse events. A third dose of inactivated SARS-CoV-2 vaccination was well tolerated and safe in MASLD patients. J. Wang et al. also reported that no serious adverse local or systemic events related to vaccines occurred in MASLD patients [34].

This study has several limitations. First, antibody levels were measured based on only one blood sample for each participant, with no continuous data collection. Dynamic changes in antibody levels could not be observed within six months. Second, COVID-19 was solely based upon patients’ reports and no other means. It remains unknown to what extent inactivated vaccination contributes to protecting MASLD patients from SARS-CoV-2 infection in the real world. We will continue to measure the new events of COVID-19 illness in MASLD patients, and compare them with normal individuals over a long duration in the future work.

In conclusion, lower antibody responses were observed in MASLD patients six months after their third dose of the inactivated vaccine than in healthy volunteers. These data may provide further assistance in monitoring patients who are more vulnerable to hypo-responsiveness to SARS-CoV-2 vaccines.

## Electronic supplementary material

Below is the link to the electronic supplementary material.

Supplementary Material 1

Supplementary Material 2

## Data Availability

The datasets used and/or analysed during the current study are available from the corresponding author on reasonable request.

## Abbreviations

COVID-19: Coronavirus Disease 2019
IQR: Interquartile Range
MASLD: Metabolic Dysfunction-Associated Steatotic Liver Disease
SARS-CoV-2: Severe Acute Respiratory Syndrome Coronavirus 2
NAb: Neutralizing Antibody
S/CO: Signal-to-cut-off ratio
