# Supplementary material for: Long-term antibody response after the third dose of inactivated SARS-CoV-2 vaccine in MASLD patients
Source: BMC Gastroenterol. 2024 Sep 30;24:329. doi: 10.1186/s12876-024-03402-9 (PMC11441169; doi:10.1186/s12876-024-03402-9)
Supplement: Supplementary file 1 — Supplementary Material 1 [file 12876_2024_3402_MOESM1_ESM.docx]

**Supplementary Table.** Factors related to IgG negativity in MASLD patients

| Characteristics | Univariate Analysis | | Multivariate Analysis | | | |
| --- | --- | --- | --- | --- | --- | --- |
|  | OR (95%CI) | P Value | | OR (95%CI) | | P Value |
| Age, y | 1.029 (1.011, 1.047) | 0.001 | | 1.03 (1.01, 1.06) | | 0.011 |
| Gender, (male, n (%)) | 1.898 (1.197, 3.011) | 0.006 | |  | |  |
| BMI, Kg/m² | 1.116 (1.047, 1.188) | 0.001 | |  | |  |
| Overweight or obesity | 1.818 (1.132, 2.921) | 0.013 | |  | |  |
| Vaccination status |  |  | |  | |  |
| Time from 2nd vaccination | 0.994 (0.988, 1.001) | 0.083 | | 0.99 (0.98, 1.00) | | 0.011 |
| Time from 3rd vaccination | 1.003 (0.999, 1.006) | 0.151 | | 1.01 (1.00, 1.01) | | 0.013 |
| MASLD severity (moderate/severe vs. mild) | 2.059 (1.026, 4.132) | 0.042 | | 2.96 (1.31, 6.72) | | 0.009 |
| PLT,10^9^ | 0.999 (0.9995, 1.002) | 0.442 | |  | |  |
| WBC,10^9^ | 0.991 (0.860, 1.142) | 0.904 | |  | |  |
| AST, U/L | 0.998 (0.985, 1.011) | 0.781 | |  | |  |
| ALT, U/L | 1.000 (0.996, 1.005) | 1.000 | |  | |  |
| GGT, U/L | 1.007 (1.000, 1.014) | 0.039 | |  | |  |
| AKP, U/L | 1.003 (0.993, 1.013) | 0.544 | |  | |  |
| ALB, g/L | 0.991 (0.901, 1.091) | 0.859 | |  | |  |
| TBIL, mmol/L | 1.003 (0.967, 1.040) | 0.879 | |  | |  |
| DBIL, mmol/L | 1.018 (0.821, 1.262) | 0.874 | |  | |  |
| BUN, mmol/L | 1.154 (0.958, 1.390) | 0.132 | |  | |  |
| Cr, μmol/L | 1.012 (0.996, 1.029) | 0.146 | |  | |  |
| GLU, mmol/L | 1.113 (0.931, 1.332) | 0.241 | |  | |  |
| TC, mmol/L | 0.977 (0.779, 1.226) | 0.844 | |  |  | |
| TG, mmol/L | 1.125 (0.900, 1.406) | 0.301 | |  |  | |
| LDL, mmol/L | 1.047 (0.781, 1.402) | 0.760 | |  |  | |
| HDL, mmol/L | 0.652 (0.330, 1.286) | 0.217 | |  |  | |
| Comorbidities, n (%) |  |  | |  |  | |
| DM, n (%) | 2.727 (1.185, 6.275) | 0.018 | |  |  | |

Abbreviations: AKP, alkaline phosphatase; ALB, albumin; ALT, alanine aminotransferase; AST, aspartate aminotransferase; BMI, body mass index; BUN, blood urea nitrogen; Cr, creatinine; DBIL, direct bilirubin; DM, diabetes mellitus; GGT, g-glutamyl transpeptidase; GLU, glucose; HDL, high-density lipoprotein; LDL, low-density lipoprotein; MASLD, metabolic dysfunction-associated steatotic liver disease; PLT, platelet; TBIL, total bilirubin; TC, total cholesterol; TG, triglyceride; WBC, white blood cell.
